# Impact of a Nurse Navigator Program on Referral Rates and Use of Fertility Preservation Among Female Cancer Patients: A 14‐Year Retrospective Cohort Study

**DOI:** 10.1002/cam4.70529

**Published:** 2025-01-30

**Authors:** Mackenzie Naert, Kimia Sorouri, Andrea Lanes, Abigail M. Kempf, Lucy Chen, Randi Goldman, Ann H. Partridge, Elizabeth Ginsburg, Serene S. Srouji, Zachary Walker

**Affiliations:** ^1^ Brigham and Women's Hospital Boston Massachusetts USA; ^2^ Harvard Medical School Boston Massachusetts USA; ^3^ Dana‐Farber Cancer Institute Boston Massachusetts USA; ^4^ University of Alberta Edmonton Alberta Canada; ^5^ Massachusetts General Hospital Boston Massachusetts USA; ^6^ Northwell New Hyde Park New York USA; ^7^ Northwell Health Fertility Manhasset New York USA

**Keywords:** fertility preservation, nurse navigator, oncofertility, referrals, utilization

## Abstract

**Introduction:**

Given the known detrimental impact of cancer treatment on fertility, fertility preservation (FP) is recommended for reproductive age patients who are newly diagnosed with cancer. However, the rate of referral to fertility specialists remains suboptimal. The objective of this study was to determine the impact of a dedicated Nurse Navigator Program (NNP) on the rate of referrals and utilization of FP services.

**Methods:**

A retrospective cohort study of all women ≥ 18 years old referred for FP consultation with a known cancer diagnosis from 2007 to 2021 at a single, large academic center was conducted. FP referrals for non‐cancer indications were excluded. Descriptive statistics were performed including comparing referrals received per 30 days and FP utilization rates pre‐NNP (October 2007–September 2013) to post‐NNP (October 2013–December 2021).

**Results:**

A total of 176 patients were included pre‐NNP and 990 patients post‐NNP. Overall, the mean age at the time of referral was 31.5 ± 6.9 years. The referral rates post‐NNP were higher among those without prior exposure to chemotherapy/radiation (0.33 pre‐NNP vs. 2.75 post‐NNP per 30 days, *p <* 0.01) and lower among those with prior exposure to chemotherapy/radiation (1.26 pre‐NNP vs. 0.70 post‐NNP per 30 days, *p* < 0.01).

**Conclusions:**

After the launch of a dedicated fertility preservation nurse navigation program at our institution, we observed a higher number of referrals for FP as well as greater use of FP overall. While not the only variable that changed during this period, this program has optimized patient care and clinical workflow at our institution and serves as a model for such improvement.

## Graphical abstract

A Nurse Navigator Program facilitates the referral pathway for patients with a cancer diagnosis interested in fertility preservation and results in higher referral and utilization rates of fertility preservation services for patients prior to receiving cancer treatment.

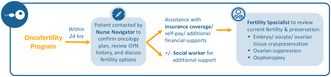

## 1 Introduction

Treatment for cancer can significantly impact ovarian reserve and function, resulting in a decline in fertility [1, 2, 3, 4, 5, 6, 7, 8]. Concerns regarding infertility have a significant negative impact on patients' well‐being and have been shown to influence treatment decisions [9]. Studies have shown that fertility‐related psychological distress has lasting impacts that persist from diagnosis to survivorship, with the potential loss of fertility as distressing as the cancer diagnosis itself for some patients [10, 11, 12]. The concept of fertility preservation (FP) encompasses several different strategies to help cancer patients retain their potential to have biological children after cancer treatment. In women, these include ovarian suppression with a GnRH agonist (for patients with breast cancer), embryo cryopreservation, oocyte cryopreservation, ovarian tissue cryopreservation and reimplantation, ovarian transposition, and fertility‐sparing surgery.

Both the American Society of Clinical Oncology (ASCO) and the American Society for Reproductive Medicine (ASRM) have published evidence‐based clinical practice guidelines on FP, which were most recently updated in 2018 and 2019, respectively. These guidelines recommend that healthcare providers caring for pediatric and reproductive‐aged women with cancer should address the possibility of infertility before treatment starts, and refer those who express interest in FP to reproductive specialists [13, 14]. Despite these guidelines and the well‐established benefit to patients, delivery of FP care remains a challenge at many levels, from counseling to referrals to utilization of care [15]. To address this challenge, institutions have increasingly instituted efforts focused on fertility counseling and preservation services [16, 17, 18, 19, 20]. However, there are limited data available regarding the impact of such programs on FP process and outcome measures including rates and timing of referrals and use of FP services. Therefore, the objective of this study was to determine the impact of a dedicated Nurse Navigator Program (NNP) initiated at our tertiary care institution on referral rates and FP utilization rates.

## 2 Materials and Methods

### 2.1 Referral Program

**FIGURE 1:**
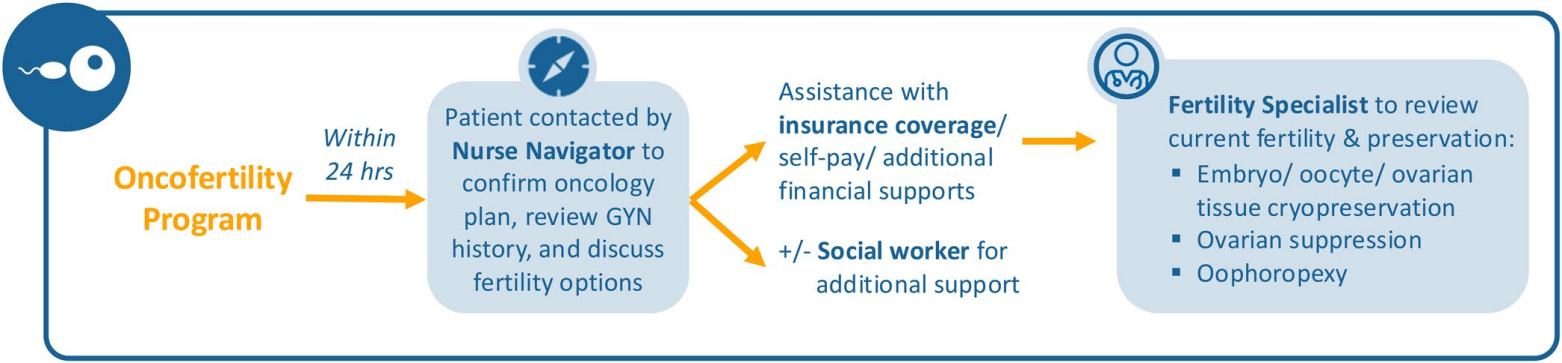
Fertility preservation referral program patient flow.

In 2013, efforts to coordinate oncofertility efforts for adult patients (age ≥ 18 years) at our academic medical center (Brigham and Women's Hospital, BWH), and the affiliated oncology center, Dana‐Farber Cancer Institute (DFCI) (collectively comprising the Dana‐Farber/Brigham and Women's Cancer Center, DF/BWCC) led to the hiring of a dedicated infertility nurse navigator to facilitate and expedite FP referrals and services for patients with new cancer diagnoses (Figure 1). In late 2013, an internal electronic referral service was implemented (initially through the intranet and later through our current shared EPIC electronic health record), allowing DF/BWCC oncologists to send electronic requests for urgent FP consultation. The electronic referral was sent directly to the nurse navigator who contacted the patient within 24 h and performed a screening telephone call. The goal of this call was to obtain their medical, obstetric, and gynecologic history, assess their understanding of the oncology treatment plan, educate them about FP options (including timing, insurance, and cost issues), and gauge their interest in proceeding with FP. After this call, if the patient chose to proceed with FP care, they were scheduled with a physician within 72 h. The nurse navigator stays closely involved throughout the process after the patient completes their consultation with the physician by expediting application processes for financial coverage (i.e., Fertile Hope), obtaining the necessary medications for FP, and educating and supporting the patient's needs during the cycle. In addition, the nurse navigator communicated the FP treatment timeline to the oncology providers to ensure prompt initiation of cancer treatment following the FP cycle, as well as communicating the outcome of the FP cycle.

Prior to the NNP, there was no formal FP referral process. Typically, the oncologist would contact a fertility specialist directly by email or telephone, and the physician would add the patient onto their busy schedule for a visit when available, often within 72 h. However, this was not well‐documented and there were concerns that patients were sometimes not referred or able to be seen in a timely fashion. Furthermore, there was concern on the part of reproductive endocrinology providers who prioritized seeing these patients. The patients' expectations and lack of understanding of what FP would entail resulted in a high proportion of patients ultimately declining services for a variety of reasons, including a lack of desire to pursue FP, concerns about the cost and timeline, and being overwhelmed. Collectively, this suggested a need for optimizing pre‐counseling and additional information and support for these patients around this highly sensitive topic.

### 2.2 Study Population and Data

We conducted a retrospective cohort study of all women ≥ 18 years old referred for FP consultation with a known cancer diagnosis from October 2007 until December 2021 at the BWH Center for Infertility and Reproductive Surgery, the primary provider of reproductive technology services of the BWH and DFCI, which together have comprised the DF/BWCC. Pediatric patients were excluded from this study. FP services for non‐cancer indications were excluded, including patients with sickle cell disease, FP for transitioning in transgender patients, and elective FP. Therefore, only FP for treatment related to gonadotoxicity was included. Patients were identified by review of an internal dataset of all patients who were referred for FP that corresponded with electronic medical record order requests for referral from October 2007 to December 2021. Those who met the inclusion criteria were separated into two groups: pre‐NNP (October 2007—September 2013) and post‐NNP (October 2013—December 2021). The cohort included some women who had already undergone chemotherapy and/or pelvic radiation before referral and potential fertility procedures (designated as prior exposure), as well as others who planned to undergo chemotherapy and/or pelvic radiation after referral and potential fertility procedures for FP (designated as without prior exposure).

Patients' electronic medical records were reviewed for demographics, cancer diagnosis, and treatment status. For subgroup analyses, patients were categorized based on age and whether they already had cancer treatment at the time of FP referral.

### 2.3 Outcomes

The main outcome measures were mean referral rate (number of referrals to a fertility specialist per 30 days), mean FP utilization rate (number of patients who underwent ovulation induction [OI] per 30 days), and type of FP services utilized (oocyte cryopreservation, embryo cryopreservation, ovarian tissue cryopreservation, and ovarian suppression with GnRH agonist (e.g., leuprolide acetate), or other services [e.g., fertility‐sparing surgery or ovarian transposition]).

### 2.4 Statistical Analysis

To evaluate the potential impact of the nurse navigator referral program, findings from the pre‐NNP and the post‐NNP period were compared. Means and standard deviations were generated for continuous variables and frequencies and proportions for categorical variables. Chi‐square tests and Fisher's exact tests were used to test for significance. An alpha of 0.05 was considered statistically significant. All statistical analyses were performed with SAS version 9.4 (Cary, NC, USA). Massachusetts General Brigham Institutional Review Board (IRB) approval was obtained (protocol #2022P000327) and was exempt from requiring written informed consent.

## 3 Results

There were a total of 1166 patients included in the study: 176 pre‐NNP and 990 post‐NNP (Table 1). The mean age at the time of referral was 31.5 ± 6.9 years. The majority of patients were White (81%), Non‐Hispanic (94%), and had private insurance (78%). The most prevalent cancer diagnoses were breast (46%), hematologic (15%), sarcoma (6%), brain (5%), or ovarian (5%) cancer. FP services utilized included OI for oocyte or embryo cryopreservation (44%), ovarian suppression with GnRH agonist (16%), and ovarian tissue cryopreservation (0.4%). Thirty percent of patients had prior cancer treatment consisting of chemotherapy and/or pelvic radiation, 55% had planned future cancer treatment, and 15% did not require chemotherapy or pelvic radiation treatment.

**TABLE 1:** Population demographic and clinical characteristics for all participants and patients referred for fertility preservation pre‐ and post‐Nurse Navigator Program (NNP).

Characteristics	All, *n* (%) (*N* = 1166)	Pre‐NNP, *n* (%) (*N* = 176)	Post‐NNP, *n* (%) (*N* = 990)
Age at referral^a^	31.5 (6.9)	32.5 (6.0)	31.3 (7.0)
Fertility preservation service			
Oocyte/embryo	515 (44.2)	85 (48.3)	430 (43.4)
Cryopreservation			
Ovarian tissue	5 (0.4)	0 (0)	5 (0.5)
Cryopreservation			
Ovarian suppression via	189 (16.2)	8 (4.6)	181 (18.3)
Lupron			
Other	1 (0.1)	1 (0.6)	0 (0)
None	456 (39.1)	82 (46.6)	374 (37.8)
Health insurance			
Public	65 (5.6)	3 (1.7)	62 (6.3)
Private	885 (75.9)	121 (68.8)	764 (77.2)
Self‐pay	174 (14.9)	28 (15.9)	146 (14.8)
None	17 (1.5)	0 (0)	146 (14.8)
Patient race			
White	920 (78.9)	143 (81.3)	777 (78.5)
Asian	90 (7.7)	14 (8.0)	76 (7.7)
Black	74 (6.4)	7 (4.0)	67 (6.8)
Other	48 (4.1)	4 (2.3)	44 (4.4)
Unknown	34 (2.9)	8 (4.6)	26 (2.6)
Ethnicity			
Hispanic/Latina	59 (5.1)	5 (2.8)	54 (5.5)
Non‐Hispanic/Latina	966 (82.9)	87 (49.4)	879 (88.8)
Unknown	141 (12.1)	84 (47.7)	57 (5.8)
Radiation			
Prior exposure	60 (5.2)	53 (30.1)	7 (0.7)
Without prior exposure	32 (2.7)	9 (5.1)	23 (2.3)
None	417 (35.8)	23 (13.1)	394 (39.8)
Missing	657 (56.4)	91 (51.7)	566 (57.2)
Chemotherapy			
Prior exposure	147 (12.6)	79 (44.9)	68 (6.9)
Without prior exposure	329 (28.2)	278 (28.1)	51 (29.0)
None	79 (6.8)	2 (1.1)	77 (7.8)
Missing	611 (52.4)	44 (25)	567 (57.3)
Chemotherapy or radiation			
Prior exposure	162 (13.9)	92 (52.3)	70 (7.1)
Without prior exposure	300 (25.7)	24 (13.6)	276 (27.9)
None	79 (6.8)	2 (1.1)	77 (7.8)
Missing	625 (53.6)	58 (33.0)	567 (57.3)

*Note:* Data reported as number (%) unless otherwise stated.

Abbreviations: *n*, number; *N*, population size; NNP, nurse navigator program; OI, ovulation induction.

^a^ Values are reported as mean (± SD) and number (%).

### 3.1 Fertility Preservation Referral Rates

For patients with prior exposure to cancer treatment consisting of chemotherapy and/or radiation, the mean rate of referrals to FP decreased after the NNP was implemented (from 1.26 referrals per 30 days to 0.70 referrals per 30 days, *p* < 0.01) (Table 2). For patients without prior exposure to chemotherapy and/or radiation, the mean rate of referrals to FP increased after the NNP was implemented (from 0.33 referrals per 30 days to 2.75 referrals per 30 days, *p* < 0.01). These changes were seen across all age groups.

**TABLE 2:** Number of referrals to a fertility specialist in total and per 30 days pre‐ and post‐Nurse Navigator Program (NNP) by age group and timing of cancer treatment.

	Pre‐NNP (*N* = 116)		Post‐NNP (*N* = 346)		*p*	
	Prior exposure	Without prior exposure	Prior exposure	Without prior exposure	Prior exposure	Without prior exposure
Referrals, *n* (%)						
Total	92 (100)	24 (100)	70 (100)	276 (100)	**< 0.01**	**0.07**
< 35	55 (59.8)	11 (45.8)	61 (87.1)	191 (69.2)		
35–37	17 (18.5)	7 (29.2)	6 (8.6)	47 (17.0)		
38–39	6 (6.5)	5 (20.8)	1 (1.4)	22 (8.0)		
40–42	9 (9.8)	1 (4.2)	1 (1.4)	10 (3.6)		
>42	5 (5.4)	0 (0)	1 (1.4)	6 (2.2)		
Referral rate^a^						
Total	1.26	0.33	0.70	2.75	**< 0.01**	**< 0.01**
< 35	0.75	0.15	0.61	1.90	0.25	**< 0.01**
35–37	0.23	0.10	0.06	0.47	**< 0.01**	**< 0.01**
38–39	0.08	0.07	0.01	0.22	**0.03**	**0.01**
40–42	0.12	0.01	0.01	0.10	**< 0.01**	**0.02**
> 42	0.07	0	0.01	0.06	0.06	—

*Note:* Bold values indicate statistical significance with *p* value < 0.05.

^a^ Per 30 days.

### 3.2 Utilization of Fertility Preservation Services

The only age group of patients with prior exposure that had a statistically significant increase in the rate of OI utilization after implementation of the NNP were patients aged < 35 years (0.37 patients per 30 days to 0.61 patients per 30 days, *p* = 0.03) (Table 2). The rate of OI utilization among all patients with prior exposure did not change after the NNP was implemented (from 0.66 patients undergoing OI per 30 days to 0.70 patients per 30 days, *p* = 0.76). For patients with no exposure, the mean rate of utilization of OI increased after the NNP was implemented (from 0.31 patients per 30 days to 2.75 patients per 30 days, *p* < 0.01). This change was seen across all age groups (Table 3).

**TABLE 3:** Utilization of fertility preservation services as the number of patients who underwent treatment in total and per 30 days pre‐ and post‐Nurse Navigator Program (NNP) by age group and timing of cancer treatment.

	Pre‐NNP (*N* = 71)		Post‐NNP (*N* = 346)		*p*	
	Prior exposure	Without prior exposure	Prior exposure	Without prior exposure	Prior exposure	Without prior exposure
Utilization of fertility preservation services, *n* (%)						
Total	48 (100)	23 (100)	70 (100)	276 (100)	**< 0.01**	0.14
< 35	27 (56.3)	11 (47.8)	61 (87.1)	191 (69.2)		
35–37	11 (22.9)	7 (30.4)	6 (8.6)	47 (17.0)		
38–39	4 (8.3)	4 (17.4)	1 (1.4)	22 (8.0)		
40–42	4 (8.3)	1 (4.4)	1 (1.4)	10 (3.6)		
> 42	2 (4.2)	0 (0)	1 (1.4)	6 (2.2)		
Rate of fertility preservation^a^						
Total	0.66	0.31	0.70	2.75	0.76	**< 0.01**
< 35	0.37	0.15	0.61	1.90	**0.03**	**< 0.01**
35–37	0.15	0.10	0.06	0.47	0.07	**< 0.01**
38–39	0.05	0.05	0.01	0.22	0.12	**< 0.01**
40–42	0.05	0.01	0.01	0.10	0.12	**0.02**
> 42	0.03	0	0.01	0.06	0.46	—

*Note:* Bold values indicate statistical significance with *p* value < 0.05.

^a^ Per 30 days.

There was a greater number of total OI cycles, as well as OI cycles for FP, at our institution during the post‐NNP time period (85/8363 cycles pre‐NNP vs. 430/11,118 cycles post‐NNP) (Table 4). This represented an increase in the percentage of FP cycles from 1.02% to 3.87% (*p* < 0.01). There were no cases of ovarian tissue freezing in the pre‐NNP cohort; however, there were 5 cases of ovarian tissue freezing in the post‐NNP group, between the years of 2015 and 2021.

**TABLE 4:** Fertility preservation (FP) cycles as proportion of total ovulation induction (OI) cycles, pre‐and post‐Nurse Navigator Program (NNP).

	Pre‐NNP	Post‐NNP	*p*
Total number of OI cycles, *n*	8363	11,118	—
Total number of FP cycles, *n*	85	430	—
Percentage of total cycles that are FP cycles, %	1.02	3.87	**< 0.01**

*Note:* Bold values indicate statistical significance with *p* value < 0.05.

## 4 Discussion

In this study, we found that the implementation of an NNP facilitated the referral pathway for patients with a cancer diagnosis interested in FP, resulting in higher referral and utilization rates. With regard to referrals, the goal of the NNP is to facilitate a timely initial contact between the patient and the Reproductive Endocrinology and Infertility (REI) team, allowing for education about the options and timeline while gauging patient's interest in proceeding with FP. The optimal population for FP services is patients who intend to but have not yet received gonadotoxic cancer treatment. It was this group of patients among whom we saw an increase in referrals following the implementation of the NNP, a marker of success. Conversely, there was a decrease in referrals received from patients who had already received cancer treatment. While of course all patients, regardless of cancer treatment status, should have access to FP, the decrease in referral rate post‐NNP could reflect a better understanding of the gonadotoxicity of treatment and the limitations of FP services for this group of patients. Alternatively, the decline in referrals in those post‐gonadotoxic therapy could also reflect the increased utilization pre‐gonadotoxic treatment in the population of patients seeking care at our institution during the timeframe of the study. The increase in pre‐treatment referrals likely also contributes to the increase in the proportion of all OI cycles intended for FP observed following the implementation of the NNP. Given the long wait times for REI appointments and the ever‐increasing demands for infertility care, focusing resources such as physician consults on patients who demonstrate interest in FP, while still providing education and specialized fertility care, reflects thoughtful use of resources. In addition, patients who have already undergone cancer treatment can be seen in a less urgent manner.

Many barriers to delivering FP care have been identified including lack of awareness, limited time available prior to chemotherapy treatment, cost for FP procedures, logistic barriers with reproductive specialties, and disagreement on who is responsible for discussing infertility risks [19, 21]. The percentage of patients who received information from their oncologists about their fertility risk and FP options vary widely, from 51% to 95% [22, 23, 24, 25, 26]. More importantly, the rate of referral to fertility specialists among eligible patients is only 9.8%–67%, far below the optimal rate of 100% [16, 22, 27]. Among all oncology centers, only 18% have an established referral pathway for FP [28]; and in pediatric oncology programs, only 36.8% have a designated FP individual or team. In one study, the presence of dedicated FP personnel was independently associated with the ability to offer oocyte or embryo cryopreservation and ovarian tissue cryopreservation [21]. Finally, one large database study including 18,781 women with cancer in the United States found that 11.7% underwent evaluation for FP and only 6.3% pursued FP procedures [29]. The NNP described here helps to tackle many of these barriers by providing a clear circle of communication between the patient, oncologist, fertility physician, and expediting care.

In response to this problem, several papers have identified key aspects of FP care models that include the implementation of a nurse or patient navigator within the fertility department [19] and having a clearly defined referral pathway [20]. With the 2013 guidelines, ASCO extended the responsibility for discussion and referral about FP to explicitly include nurses [30]. One systematic review evaluating the nurse's role in FP found that nurses are often well‐positioned to improve FP care given their training, expertise, and unique relationship with patients [31]. Despite this opportunity, nurses currently have a minimal role because of provider, institutional, and patient‐related barriers [31, 32, 33, 34, 35]. Our study supports these findings, showing that a dedicated nurse navigator plays a crucial role in FP care.

Additionally, studies show that the impact of infertility on quality of life is even greater for patients who did not receive adequate information prior to treatment initiation and that simply receiving counseling regarding FP has positive psychological benefits [11, 12]. One systematic review found that the majority of women who received FP counseling reported that the counseling alone improved coping with the potential loss of fertility, minimized long‐term regret and dissatisfaction regarding fertility, and improved their physical quality of life [36]. By having a system in place to ensure all patients receive FP counseling, the NNP has the potential to significantly help patients' psychological well‐being.

There are several limitations of this study, including the retrospective design and lack of a control group. The study focused on the REI nurse navigator's role in the referral process. The referral process starts with the referral from the oncologist, and there are multiple factors from an oncology perspective that likely contributed to the increase in referrals and utilization of FP services following the implementation of the NNP that were not captured in our data. For example, external factors, such as the increasing awareness and improved communication regarding fertility in the world, as well as the improvements in available data for the safety and efficacy of ART in this setting could have impacted the outcomes of our study. Additionally, internal factors within DFCI to increase referrals for FP may have affected our outcomes. Of note, the study was conducted in a state that mandates fertility coverage by private insurance. As most patients included in this study reported private insurance (76%), this limits the generalizability of this study. Moreover, as the study only included patients with a new diagnosis of cancer who were referred through the NNP by an oncologist seeing the patient for active treatment of cancer, it is possible that patients had a prior childhood cancer that was not accounted for (e.g., breast cancer secondary to mantle radiation for childhood hematologic malignancy). Given that this is a retrospective study, we were only able to assess the patients who were captured in our referral system and limited by the information recorded, resulting in a high proportion of missing exposure data. Therefore, our conclusions pertaining to referral rates or utilization rates may be affected by selection bias and not truly reflect the rates for all oncologic patients. Separately, we were unable to stratify by the type of chemotherapy that patients received. Different types of chemotherapy, as well as different durations and doses, have varying impacts on ovarian reserve. Similarly, we did not collect information on cancer therapies beyond radiation and chemotherapy, such as targeted therapy and immunotherapy. In patients who had prior cancer treatment, we did not examine rates of residual ovarian function. Given that men are six times more likely to separate or divorce their spouse with a new diagnosis of cancer, patients undergoing FP are routinely recommended to bank oocytes instead of embryos [37]. Our referral database amalgamated oocyte and embryo cryopreservation thus we were not able to separate these to further assess nuances within oocyte versus embryo banking.

This study demonstrated that an NNP facilitates the referral pathway for patients with a cancer diagnosis interested in FP, resulting in higher referral rates and utilization rates of FP services for eligible patients prior to receiving cancer treatment at a single institution. In a model of care where patients have access to a fertility clinic that is partnered with the institution providing cancer care, the ideal NNP would include follow‐up with the oncology team to report data on patients that were referred to promote continued referrals or identify disparities in which patients are referred. This would also allow programs to keep track of fertility preservation services and optimize referral rates and additionally could create internal key performance indicators to ensure that patients are being referred if they meet criteria. For example, the nurse navigator could make sure oncologists were aware of the FP program and how to utilize the service effectively, while also obtaining demographic information from oncology services to ensure that the appropriate patients who qualify for FP counseling are being referred. The nurse would then track each oncology team's referral rate and follow‐up to discuss outcomes and areas for improvement. As this integrated and comprehensive model of care is most commonly seen at high‐resource centers, on a systems level, we encourage professional associations to provide educational opportunities to support community‐based fertility clinics to become comfortable with providing FP services and to encourage comprehensive oncofertility networks in the community through partnerships between cancer centers and fertility clinics. For example, community‐based cancer centers could have a contact person (e.g., Nurse Practitioner or Advanced Practice Provider) at local fertility centers to expedite referral processes to allow patients to be seen via telehealth, or in‐person, for consultation within 24–48 h of referral. In addition, professional associations should recommend that fertility clinics that are already providing FP services have a dedicated nurse navigator to optimize patient care and clinical workflow. By doing so, we can help provide important counseling and care to women during an incredibly stressful period of their lives, improve access to care, and optimize the utilization of resources.

## 5 Conclusion

After the launch of a dedicated fertility preservation nurse navigation program at our institution, we observed a higher number of referrals for FP including an apparent shift in the timing of referrals to earlier in their cancer care (prior to treatment), as well as greater use of FP overall. While not the only variable that changed during this period, this program has optimized patient care and clinical workflow at our institution and serves as a model for such improvement.

## Author Contributions

**Mackenzie Naert:** data curation (equal), investigation (equal), writing – original draft (equal). **Kimia Sorouri:** data curation (equal), formal analysis (equal), investigation (equal), writing – original draft (equal), writing – review and editing (equal). **Andrea Lanes:** data curation (equal), formal analysis (equal), methodology (equal), writing – review and editing (equal). **Abigail M. Kempf:** data curation (equal), formal analysis (equal), investigation (equal), writing – review and editing (equal). **Lucy Chen:** data curation (equal), formal analysis (equal), investigation (equal), writing – review and editing (equal). **Randi Goldman:** conceptualization (equal), writing – review and editing (equal). **Ann H. Partridge:** writing – review and editing (equal). **Elizabeth Ginsburg:** conceptualization (equal), data curation (equal), formal analysis (equal), methodology (equal), supervision (equal), writing – review and editing (equal). **Serene S. Srouji:** conceptualization (equal), investigation (equal), writing – review and editing (equal). **Zachary Walker:** conceptualization (equal), data curation (equal), formal analysis (equal), investigation (equal), methodology (equal), project administration (equal), supervision (equal), writing – original draft (equal), writing – review and editing (equal).

## Conflicts of Interest

The authors declare no conflicts of interest.

## Data Availability

The data that support the findings of this study are available on request from the corresponding author. The data are not publicly available due to privacy or ethical restrictions.

## References

[1] W. H. B. Wallace , A. B. Thomson , F. Saran , and T. W. Kelsey , “Predicting Age of Ovarian Failure After Radiation to a Field That Includes the Ovaries,” International Journal of Radiation Oncology, Biology, Physics 62, no. 3 (2005): 738–744. 15936554 10.1016/j.ijrobp.2004.11.038

[2] J. Y. Wo and A. N. Viswanathan , “Impact of Radiotherapy on Fertility, Pregnancy, and Neonatal Outcomes in Female Cancer Patients,” International Journal of Radiation Oncology, Biology, Physics 73, no. 5 (2009): 1304–1312. 19306747 10.1016/j.ijrobp.2008.12.016 PMC2865903

[3] N. Spears , F. Lopes , A. Stefansdottir , et al., “Ovarian Damage From Chemotherapy and Current Approaches to Its Protection,” Human Reproduction Update 25, no. 6 (2019): 673–693. 31600388 10.1093/humupd/dmz027 PMC6847836

[4] W. van Dorp , R. Haupt , R. A. Anderson , et al., “Reproductive Function and Outcomes in Female Survivors of Childhood, Adolescent, and Young Adult Cancer: A Review,” Journal of Clinical Oncology 36, no. 21 (2018): 2169–2180. 29874135 10.1200/JCO.2017.76.3441 PMC7098836

[5] J. M. Letourneau , E. E. Ebbel , P. P. Katz , et al., “Acute Ovarian Failure Underestimates Age‐Specific Reproductive Impairment for Young Women Undergoing Chemotherapy for Cancer,” Cancer 118, no. 7 (2012): 1933–1939. 21850728 10.1002/cncr.26403 PMC3220922

[6] T. Schuurman , S. Zilver , S. Samuels , et al., “Fertility‐Sparing Surgery in Gynecologic Cancer: A Systematic Review,” Cancers 13, no. 5 (2021): 1008. 33670929 10.3390/cancers13051008 PMC7975326

[7] E. Somigliana , G. Ragni , F. Benedetti , R. Borroni , W. Vegetti , and P. G. Crosignani , “Does Laparoscopic Excision of Endometriotic Ovarian Cysts Significantly Affect Ovarian Reserve?,” Insights From IVF Cycles. Human Reproduction 18, no. 11 (2003): 2450–2453. 14585900 10.1093/humrep/deg432

[8] X. Ye , Y. Yang , and X. Sun , “A Retrospective Analysis of the Effect of Salpingectomy on Serum AntiMüllerian Hormone Level and Ovarian Reserve,” American Journal of Obstetrics and Gynecology 212, no. 1 (2015): 53. e10. 10.1016/j.ajog.2014.07.027 25046807

[9] A. H. Partridge , S. Gelber , J. Peppercorn , et al., “Web‐Based Survey of Fertility Issues in Young Women With Breast Cancer,” Journal of Clinical Oncology 22 (2004): 4174–4183. 15483028 10.1200/JCO.2004.01.159

[10] S. Tschudin and J. Bitzer , “Psychological Aspects of Fertility Preservation in Men and Women Affected by Cancer and Other Life‐Threatening Diseases,” Human Reproduction Update 15, no. 5 (2009): 587–597. 19433413 10.1093/humupd/dmp015

[11] J. Goossens , I. Delbaere , D. Beeckman , S. Verhaeghe , and A. Van Hecke , “Communication Difficulties and the Experience of Loneliness in Patients With Cancer Dealing With Fertility Issues: A Qualitative Study,” Oncology Nursing Forum 42 (2015): 34–43. 25542319 10.1188/15.ONF.34-43

[12] S. T. Vadaparampil , N. M. Hutchins , and G. P. Quinn , “Reproductive Health in the Adolescent and Young Adult Cancer Patient: An Innovative Training Program for Oncology Nurses,” Journal of Cancer Education 28, no. 1 (2013): 197–208. 23225072 10.1007/s13187-012-0435-z PMC3610840

[13] K. Oktay , B. E. Harvey , and A. W. Loren , “Fertility Preservation in Patients With Cancer: ASCO Clinical Practice Guideline Update Summary,” Journal of Oncology Practice 14 (2018): 381–385. 29768110 10.1200/JOP.18.00160

[14] Practice Committee of the American Society for Reproductive Medicine , “Fertility Preservation in Patients Undergoing Gonadotoxic Therapy or Gonadectomy: A Committee Opinion,” Fertility and Sterility 112, no. 6 (2019): 1022–1033. 31843073 10.1016/j.fertnstert.2019.09.013

[15] A. Anazodo , P. Laws , S. Logan , et al., “How Can We Improve Oncofertility Care for Patients? A Systematic Scoping Review of Current International Practice and Models of Care,” Human Reproduction Update 25, no. 2 (2019): 159–179. 30462263 10.1093/humupd/dmy038 PMC6390168

[16] G. P. Quinn , S. T. Vadaparampil , J. H. Lee , et al., “Physician Referral for Fertility Preservation in Oncology Patients: A National Study of Practice Behaviors,” Journal of Clinical Oncology 27, no. 35 (2009): 5952–5957. 19826115 10.1200/JCO.2009.23.0250

[17] E. M. Mobley , G. L. Ryan , A. E. Sparks , V. Monga , and W. W. Terry , “Factors Impacting Fertility Preservation in Adolescents and Young Adults With Cancer: A Retrospective Study,” Journal of Adolescent and Young Adult Oncology 9, no. 2 (2020): 208–221. 31651207 10.1089/jayao.2019.0100 PMC7360112

[18] C. A. Carlson , T. F. Kolon , P. Mattei , et al., “Developing a Hospital‐Wide Fertility Preservation Service for Pediatric and Young Adult Patients,” Journal of Adolescent Health 61, no. 5 (2017): 571–576. 10.1016/j.jadohealth.2017.07.008 28917444

[19] M. van den Berg , Ö. Baysal , W. L. D. M. Nelen , D. D. M. Braat , C. C. M. Beerendonk , and R. P. M. G. Hermens , “Professionals' Barriers in Female Oncofertility Care and Strategies for Improvement,” Human Reproduction (Oxford, England) 34, no. 6 (2019): 1074–1082. 31111876 10.1093/humrep/dez062

[20] A. Anazodo , P. Laws , S. Logan , et al., “The Development of an International Oncofertility Competency Framework: A Model to Increase Oncofertility Implementation,” Oncologist 24, no. 12 (2019): e1450–e1459. 31147490 10.1634/theoncologist.2019-0043 PMC6975957

[21] N. N. Frederick , J. L. Klosky , L. R. Meacham , et al., “Infrastructure of Fertility Preservation Services for Pediatric Cancer Patients: A Report From the Children's Oncology Group,” JCO Oncology Practice 18, no. 3 (2022): e325–e333. 34709943 10.1200/OP.21.00275 PMC8932529

[22] E. Adams , E. Hill , and E. Watson , “Fertility Preservation in Cancer Survivors: A National Survey of oncologists' Current Knowledge, Practice and Attitudes,” British Journal of Cancer 108, no. 8 (2013): 1602–1615. 23579214 10.1038/bjc.2013.139 PMC3668471

[23] E. J. Forman , C. K. Anders , and M. A. Behera , “A Nationwide Survey of Oncologists Regarding Treatment‐Related Infertility and Fertility Preservation in Female Cancer Patients,” Fertility and Sterility 94, no. 5 (2010): 1652–1656. 19945099 10.1016/j.fertnstert.2009.10.008

[24] L. A. Louwé , A. M. Stiggelbout , A. Overbeek , C. G. J. M. Hilders , M. H. van den Berg , and E. Wendel , “Factors Associated With Frequency of Discussion of or Referral for Counselling About Fertility Issues in Female Cancer Patients,” European Journal of Cancer Care 27, no. 1 (2018): e12602. 10.1111/ecc.12602 27774666

[25] E. E. Niemasik , J. Letourneau , D. Dohan , et al., “Patient Perceptions of Reproductive Health Counseling at the Time of Cancer Diagnosis: A Qualitative Study of Female California Cancer Survivors,” Journal of Cancer Survivorship: Research and Practice 6, no. 3 (2012): 324–332. 22752834 10.1007/s11764-012-0227-9

[26] J. M. Letourneau , E. E. Ebbel , P. P. Katz , et al., “Pretreatment Fertility Counseling and Fertility Preservation Improve Quality of Life in Reproductive Age Women With Cancer,” Cancer 118, no. 6 (2012): 1710–1717. 21887678 10.1002/cncr.26459 PMC3235264

[27] L. Bastings , O. Baysal , C. C. M. Beerendonk , D. D. M. Braat , and W. L. D. M. Nelen , “Referral for Fertility Preservation Counselling in Female Cancer Patients,” Human Reproduction (Oxford, England) 29, no. 10 (2014): 2228–2237. 25069500 10.1093/humrep/deu186

[28] E. Warner , S. Yee , E. Kennedy , et al., “Oncofertility Knowledge, Attitudes, and Practices of Canadian Breast Surgeons,” Annals of Surgical Oncology 23, no. 12 (2016): 3850–3859. 27431414 10.1245/s10434-016-5423-9

[29] J. Selter , Y. Huang , L. C. G. Becht , et al., “Use of Fertility Preservation Services in Female Reproductive‐Aged Cancer Patients,” American Journal of Obstetrics and Gynecology 221, no. 4 (2019): 328.e1–328.e16. 10.1016/j.ajog.2019.05.009 31108063

[30] A. W. Loren , P. B. Mangu , L. N. Beck , et al., “Fertility Preservation for Patients With Cancer: American Society of Clinical Oncology Clinical Practice Guideline Update,” Journal of Clinical Oncology 31, no. 19 (2013): 2500–2510. 23715580 10.1200/JCO.2013.49.2678 PMC5321083

[31] C. Crespi , L. Adams , T. F. Gray , and D. R. Azizoddin , “An Integrative Review of the Role of Nurses in Fertility Preservation for Adolescents and Young Adults With Cancer,” Oncology Nursing Forum 48, no. 5 (2021): 491–505. 34411081 10.1188/21.ONF.491-505

[32] L. King , G. P. Quinn , S. T. Vadaparampil , et al., “Oncology Nurses' Perceptions of Barriers to Discussion of Fertility Preservation With Patients With Cancer,” Clinical Journal of Oncology Nursing 12, no. 3 (2008): 467–476. 18515245 10.1188/08.CJON.467-476

[33] A. Nobel Murray , J. C. Chrisler , and M. L. Robbins , “Adolescents and Young Adults With Cancer: Oncology Nurses Report Attitudes and Barriers to Discussing Fertility Preservation,” Clinical Journal of Oncology Nursing 20, no. 4 (2016): E93–E99. 27441525 10.1188/16.CJON.E93-E99

[34] W. Norton and E. Wright , “Barriers and Facilitators to Fertility‐Related Discussions With Teenagers and Young Adults With Cancer: Nurses' Experiences,” Journal of Adolescent and Young Adult Oncology 9, no. 4 (2020): 481–489. 32155354 10.1089/jayao.2019.0092

[35] S. T. Vadaparampil , H. Clayton , G. P. Quinn , L. M. King , M. Nieder , and C. Wilson , “Pediatric Oncology Nurses' Attitudes Related to Discussing Fertility Preservation With Pediatric Cancer Patients and Their Families,” Journal of Pediatric Oncology Nursing 24, no. 5 (2007): 255–263. 17827491 10.1177/1043454207303878

[36] N. A. Deshpande , I. M. Braun , and F. L. Meyer , “Impact of Fertility Preservation Counseling and Treatment on Psychological Outcomes Among Women With Cancer: A Systematic Review,” Cancer 121, no. 22 (2015): 3938–3947. 26264701 10.1002/cncr.29637

[37] M. J. Glantz , M. C. Chamberlain , Q. Liu , et al., “Gender Disparity in the Rate of Partner Abandonment in Patients With Serious Medical Illness,” Cancer 115, no. 22 (2009): 5237–5242. 19645027 10.1002/cncr.24577

